# Synthetic PreImplantation Factor (sPIF) reduces inflammation and prevents preterm birth

**DOI:** 10.1371/journal.pone.0232493

**Published:** 2020-06-08

**Authors:** Marialuigia Spinelli, Céline Boucard, Fiorella Di Nicuolo, Valerie Haesler, Roberta Castellani, Alfredo Pontecorvi, Giovanni Scambia, Chiara Granieri, Eytan R. Barnea, Daniel Surbek, Martin Mueller, Nicoletta Di Simone

**Affiliations:** 1 Department of Obstetrics and Gynecology and Department of Biomedical Research, University Hospital Bern, University of Bern, Bern, Switzerland; 2 Università Cattolica del Sacro Cuore, Istituto di Clinica Ostetrica e Ginecologica, Roma, Italia; 3 International Scientific Institute Paolo VI, Università Cattolica Del Sacro Cuore, A. Gemelli Universitary Hospital, Rome, Italia; 4 U.O.C di Endocrinologia e Diabetologia, Dipartimento di Scienze Gastroenterologiche, Endocrino-Metaboliche e Nefro-Urologiche, Fondazione Policlinico Universitario A. Gemelli IRCCS, Roma, Italia; 5 U.O.C. di Ginecologia Oncologica, Dipartimento di Scienze della Salute della Donna, del Bambino e di Sanità Pubblica, Fondazione Policlinico Universitario A. Gemelli IRCCS, Roma, Italia; 6 The Society for The Investigation of Early Pregnancy (SIEP), Cherry Hill, NJ, United States of America; 7 BioIncept LLC, Cherry Hill, NJ, United States of America; 8 Dipartimento di Scienze della Salute della Donna e del Bambino, Fondazione Policlinico Universitario A. Gemelli IRCCS, U.O.C. di Ostetricia e Patologia Ostetrica, Roma, Italia; John Hunter Hospital, AUSTRALIA

## Abstract

Preterm birth (PTB) is the leading cause of neonatal morbidity and mortality and spontaneous PTB is a major contributor. The preceding inflammation/infection contributes not only to spontaneous PTB but is associated with neonatal morbidities including impaired brain development. Therefore, control of exaggerated immune response during pregnancy is an attractive strategy. A potential candidate is synthetic PreImplantation Factor (sPIF) as sPIF prevents inflammatory induced fetal loss and has neuroprotective properties. Here, we tested maternal sPIF prophylaxis in pregnant mice subjected to a lipopolysaccharides (LPS) insult, which results in PTB. Additionally, we evaluated sPIF effects in placental and microglial cell lines. Maternal sPIF application reduced the LPS induced PTB rate significantly. Consequently, sPIF reduced microglial activation (Iba-1 positive cells) and preserved neuronal migration (Cux-2 positive cells) in fetal brains. In fetal brain lysates sPIF decreased IL-6 and INFγ concentrations. In-vitro, sPIF reduced Iba1 and TNFα expression in microglial cells and reduced the expression of pro-apoptotic (*Bad* and *Bax*) and inflammatory (*IL-6* and *NLRP4*) genes in placental cell lines. Together, maternal sPIF prophylaxis prevents PTB in part by controlling exaggerated immune response. Given the sPIF`FDA Fast Track approval in non-pregnant subjects, we envision sPIF therapy in pregnancy.

## Introduction

Preterm birth (PTB) is currently the leading cause of childhood mortality under the age of five [1]. Spontaneous PTB accounts for approximately 70% of PTBs and strongly associated with inflammation/infection cascade [2]. Recent evidence suggest that the overall shift in the inflammatory environment, rather than the presence of individual microbial communities during microbiome disruption, contributes to spontaneous PTB [3]. In line with this notion, anti-microbial treatments have shown limited efficacy in reducing PTB risk [4]. Interestingly, an inflammatory insult contributes not only to the PTB risk but also to the risk of fetal mortality and morbidities. For example, chorioamnionitis is associated with higher rate of cerebral palsy, necrotising enterocolitis, and patent ductus arteriosus of the fetus [5–7]. Survivor of PTB have a higher incidence of cognitive and/or behavioral deficits such as sensory, learning/memory, language, attention deficits and hyperactivity, and autism spectrum disorder [8,9]. Together, proper control of an exaggerated inflammatory response should not only reduce the PTB incidence but also impact fetal morbidities such as brain injury [10,11].

In pregnancy, the immune system is complex and the set of pathogen recognition receptors: toll-like receptors (TLRs) are essential [4,12]. TLRs are activated by pathogen associated molecular patterns (PAMPs) resulting in activation of inflammatory cascades [13,14]. The bacteria derived major pro-inflammatory antigen lipopolysaccharide (LPS) serve as PAMPs and not surprisingly, the LPS-induced inflammation animal model is a frequently used and well-described model to study inflammation induced PTB [15]. Furthermore, LPS induces macrophage-derived tumor necrosis factor (TNF)-α production and interferon (INF)-γ secretion [16], which result in release of feto-toxic cytokines contributing to fetal long-term morbidities [17,18]. We postulate that PTB targeted prevention requires maternal and fetal immune control. A potent candidate is PreImplantation Factor (PIF) since PIF shapes the immune system during pregnancy [19,20].

PIF is a small 15-amino acid pregnancy derived peptide, which is secreted by the trophoblast/embryo [21,22]. Trophoblast invasion and endometrial receptivity resulting in proper embryo development and finally a favorable pregnancy outcome are in part the result of PIF secretion [21–29]. Mechanistically, PIF modulates immune responses [30,31], while reducing oxidative stress and protein misfolding [28,29]. Not surprisingly, a synthetic version of PIF (PIF analog: sPIF) was successfully tested in animal models of multiple immune disorders [32–38] and received a FAST-Track FDA approval (autoimmune diseases of non-pregnant subjects—clinicaltrials.gov, NCT02239562). Together, sPIF is a pregnancy derived peptide exerting immune-modulatory properties, which at least in part were TLR4 mediated [35,36]. As TLRs are essential during inflammatory PTB and sPIF modulates inflammation during pregnancy, we tested whether maternal sPIF pre-treatment is able to reduce the incidence of PTB and resulting immune responses.

## Materials and methods

### Animals and treatments

We synthesized synthetic PIF_15_ (MVRIKPGSANKPSDD) by solid-phase peptide synthesis (Peptide Synthesizer, Applied Biosystems) employing Fmoc (9-fluorenylmethoxycarbonyl) chemistry at Bio-Synthesis, Inc. (Lewisville, TX, USA) as previously published [20]. Briefly, we carried out final purification by reversed-phase HPLC and we verified the identity by matrix-assisted laser desorption/ionization time-of-flight mass spectrometry (amino acid analysis at >95% purity). sPIF was used for all experiments.

In order to mimic inflammatory PTB we used female Swiss mice (7–8 weeks old), which were paired with adult Swiss mice (n = 55) as previously published [20]. Briefly, we considered day 0 of gestation as the day of appearance of post-coitum vaginal plug. We housed animals in accordance with Ethics Committee and Veterinary Department guidelines. Prior to surgery, we assured acclimatization of all animals to the laboratory environment and we provided animals with food and water ad libitum. We used controlled conditions of light (12h light/12h dark) and temperature (23–25°C) for animal housing. We followed aseptic rodent survival surgery guidelines. We used 4 groups (n = 55 total) in our experimental protocol. We treated two groups of pregnant mice (14 each) with sPIF (1μg/g mouse /day) using micro-osmotic pumps from day 0 until day 14 of gestation. The other two groups received phosphate buffered solution (PBS—200 μl; n = 27) from day 0 until day 14 of gestation. On day 14 of gestation, half of each group was injected with LPS (from Escherichia Coli serotype 0111:B4; Sigma-Aldrich, St Louis, MA USA; intra-peritoneum 300 μg/kg mouse in 200 μl PBS) or PBS (200 μl). Thus, we investigated the following 4 groups: Control (n = 14), sPIF (n = 14), LPS (n = 13), and LPS+sPIF (n = 14). Notably, in multiple animal studies the dosage of synthetic PIF was used previously [20,32,33,35,36,38]. We sacrificed mice on day 16 of pregnancy and dissected the uteri and fetuses. Briefly, pregnant mice were anesthetized via intraperitoneal injection of Ketamine and Xylazine (ketamine 80–100 mg/kg, xylazine 10–12.5 mg/kg) and sacrificed by cervical dislocation, the uteri were dissected and embryos were harvested. All the embryos were processed while alive embryos were immediately anesthetized by hypothermia (placing them on ice in a Petri plate). Embryos were decapitated with scissors and half of the brain tissue was removed and stored in formalin (10% neutral-buffered solution) or (the other half) frozen at –80° C for subsequent experiments.

We implanted micro-osmotic pumps as previously performed [20]. Briefly, we used intraperitoneal injection of Ketamine and Xylazine (ketamine 80–100 mg/kg, xylazine 10–12.5 mg/kg) in mice and once we detected loss of the righting reflex we proceeded with surgery. We implanted pumps subcutaneously on the back of mice by making a small cut in the mid-scapulary region. After implantation the incision was closed with wound clip. After recovery from anesthesia, we monitored mice for several signs including bleeding, discomfort, or pain. If necessary, we used local anesthesia (lidocaine, 4 mg/kg, 0.4 ml/kg of a 1% solution).

Importantly, all used procedures followed the requirements of Commission Directive 86/609/EEC, which concern the protection of animals used for experimental and other scientific purposes. We acquired local ethical committee approval on preclinical studies [n° 5647/14 (A13 D)] from Universita`Cattolica del Sacro Cuore Roma, Italy.

### ELISA assay

Once brain tissue was collected, we dissected fetal brains and measured cytokines in those lysates (3 brains per pregnant animal pooled; n = 6–7 per group) by an enzyme-linked immunoassay (ELISA) according to manufacturer’s instructions (USCN Life Science Inc. and Cloud-Clone Corp. Houston, TX, USA). Briefly, brain tissues were collected, washed with PBS, minced and lysed using Cell lysis Buffer (Cell Signaling Technology, Inc., Danvers, MA, USA) in the presence of protease inhibitors (Roche Diagnositics, Indianapolis, IN, USA), then were sonicated briefly and centrifuged for 20 minutes at 14000 x g at 4°C. We measured the proinflammatory cytokines IL-6 and INF-γ as previously performed [20]. Briefly, we added samples or standard (100 μl) to each well coated with monoclonal anti-cytokine antibody. We incubated at 37°C for 2h, and washed the wells prior to incubation with a specific enzyme-linked polyclonal antibody, horseradish peroxidase. Then, we added tetramethylbenzidine substrate solution to each well, and the color developed in proportion to the amount of the proteins bound in the initial step. We used the Titertek Multiscan plus Mk II plate reader (ICN Flow Laboratories, Irvine, CA) to measure the absorbance at wavelengths of 450 nm.

### Placental and microglial cell culture

BV-2 microglia and BeWo placental culture: We purchased the semi-adherent mouse cell line BV-2 (ATL03001) from Banca Biologica e Cell Factory, Genoa, Italy. We used Roswell Park Memorial Institute (RPMI) 1640 with 10% FBS, 2 mmol/l GlutaMAX^™^, 100 units/ml penicillin, and 100 mg/ml streptomycin for cell expansion. We used mechanical vibrations and flushing with PBS in cells to detached the from culture plates. BeWo human choriocarcinoma cell lines were kindly provided by Prof. Christiane Albrecht (University of Bern) and cultured in expansion medium consisting of Dulbecco’s modified Eagle’s medium (DMEM)/F12 supplemented with 10% fetal bovine serum (FBS). In order to stimulate BV-2 and BeWo cells with LPS (or use sPIF), we seeded cells at density of 12’500 cells/cm2 or 18'000 cells/cm2 prior to incubation with LPS. We used 100 ng/ml LPS (Sigma-Aldrich) for 6h (BeWo) and 24 h (BV2) and after stimulation, we treated cells with sPIF (concentrations: 100, 200, or 300 nM for 4 hours). Cells cultured without the addition of sPIF served as controls.

Cell counts: We collected media from BV2 cell plates, and adherent BV2 cells through trypsinization. Both media and cells were centrifuged at 2500 rpm for 5 min at 4°C. The supernatant was removed and the cell pellet was resuspended in 1 ml of media. A small aliquot of the media was diluted with PBS and mixed with trypan blue exclusion dye. Viable and nonviable cells were counted on a hemocytometer.

### Gene quantification by real-time polymerase chain reaction (RT-PCR)

We used the QIAshredder and the Allprep DNA/RNA/Protein Mini Kit according to the manufacturer’s protocol (Qiagen, Hilden, Germany) for RNA isolation. Briefly, RNA concentration was measured using a NanoVue Plus^™^ spectrophotometer (Biochrom, Holliston, MA, USA). The RNA purity was assured by measuring the 260 nm/280 nm ratio. We considered a ratio between 1.8 and 2.1 as pure and high-quality RNA. Up to 3 μg RNA reverse transcribed was used in combination with the SuperScript III First-Strand Synthesis System (Thermo Fisher Scientific). Gene expression in cells was quantified using real-time reverse transcription polymerase chain reaction (RT-PCR). Following gene were tested: Iba1, TNFα, BAD, BAX, IL6 and NLRP4 by real-time RT-PCR. We used following PCR cycling program: 2 min at 50°C followed by 10 min at 95°C, and finally 45 cycles of 15 s at 95°C and 1 min at 60°C. We used a QuantStudio^™^ 7 Flex Real-Time PCR System (Thermo Fisher Scientific). For housekeeping gene glyceraldehyde-3-phosphate dehydrogenase as endogenous control was used. Notably, we adopted primer and probe sequences using RT Primer Database and used the QuantStudio^™^ Real-Time PCR software (Thermo Fisher Scientific) for analysis. We calculated gene expression using the 2−ΔΔCt method relative to untreated cells. We used following primers:

1. Iba1 (GE assay ID: Rn01525937_g1, Thermo Fisher Scientific, US)
2. TNFα (GE assay ID: Dr03126850_m1, Thermo Fisher Scientific, US)
3. BAD (GE assay ID: Hs00188930_m1, Thermo Fisher Scientific, US)
4. BAX (GE assay ID: Hs00180269_m1, Thermo Fisher Scientific, US)
5. IL6 (GE assay ID: Hs00174131_m1, Thermo Fisher Scientific, US)
6. NLRP4 (GE assay ID: Hs00370499_m1, Thermo Fisher Scientific, US)

### Immunohistochemistry

We used a set of fetal brains for Elisa testing (see above) and the other fetal brains for immunohistochemistry [35,36]. Briefly, after brain removal we fixed the tissue in formaldehyde solution (4%) for 2–4 hours at RT followed by 4°C for a total time of 24–48 hours. Fixed brains were embedded in paraffin and finally sectioned into 7μm slices. Following a de-paraffinization procedure, we used a pressure cooker for 12 minutes and retrieved the target citrate buffer (10 mM; pH 6.0). We used 0.1% Tween-20/PBS for washing and 10% goat serum/1% bovine albumin/1% Triton-x/PBS for blocking slides. We incubated slides overnight: first incubation at 4°C with mouse monoclonal antibodies specific for the Cut Like Homeobox 2 antigen (Cux2, Bioss, BS-11832R, 1:100) or ionized calcium-binding adapter molecule 1 (Iba1, Abcam, AB5076, 1:100). Following first antibody incubation, we washed the slides in 0.1% Tween-20/PBS (3x 5min). To highlight both microglial cells and progenitor neuronal cells, slides were incubated for one hour at RT with peroxidase-labeled secondary antibody (DAKO, Glostrup, Denmark, 1:100), as a second antibody, then washed in PBS (4x5 min). Finally we used DAB+ chromogen application in buffer substrate according to the manufacturer’s instructions (DAKO EnVision+ System-HRP (DAB), K4007). We rinsed the slides in dH2O. We used 11 minutes Cresyl violet (Nissl body staining for neuronal structure and gross brain morphology) for counterstaining and a series of ethanol baths (95% >100%) and xylene for dehydration. Slides were mounted with Eukitt (Sigma-Aldrich, St. Louis, MO).

Assessment of neuronal and microglial positive cells were performed in the region of interest (ROI). We defined ROI as cortical area between the rhinal sulcus and the cingulum (CC; contains cerebral white matter) and developing dentate gyrus germinal matrix (DGm) as injury in these regions cause distinctive neuropathological alterations [35,39–42]. All images of immunohistochemical stainings were obtained with a Zeiss based microscope (Pannoramic, 3CCD camera Hitachi HV-F22CL 1,4MP and Zeiss AxioCam MRm monochrome camera) equipped with a digital camera. We used 40× or 60× objective to acquire images for ROI evaluation. Six consecutive coronal sections per animal and for each specific immunostaining were acquired by an independent observer blinded to the experimental conditions. We analyzed and reconstructed the images using Image J (US National Institutes of Health).

### Quantification and statistical analysis

We performed all quantifications including the manual cell counts in a blinded manner. We counted Cux2 and Iba1 positive cells in the ROI (see above) as previously reported [40,43,44]. We determined the number of cells by unbiased counting of positive cells [43].

Statistical Analysis: We present the results as the mean ± standard deviation (mean±SD). We calculated the PTB significance using Chi-square test. We used one-way analysis of variance (ANOVA) followed by a post–hoc test (Bonferroni test) for additional analysis. We determined statistical significance at p<0.05.

## Results

### Synthetic PIF prevents PTB and reduces inflammation (in-vitro)

**Fig 1:**
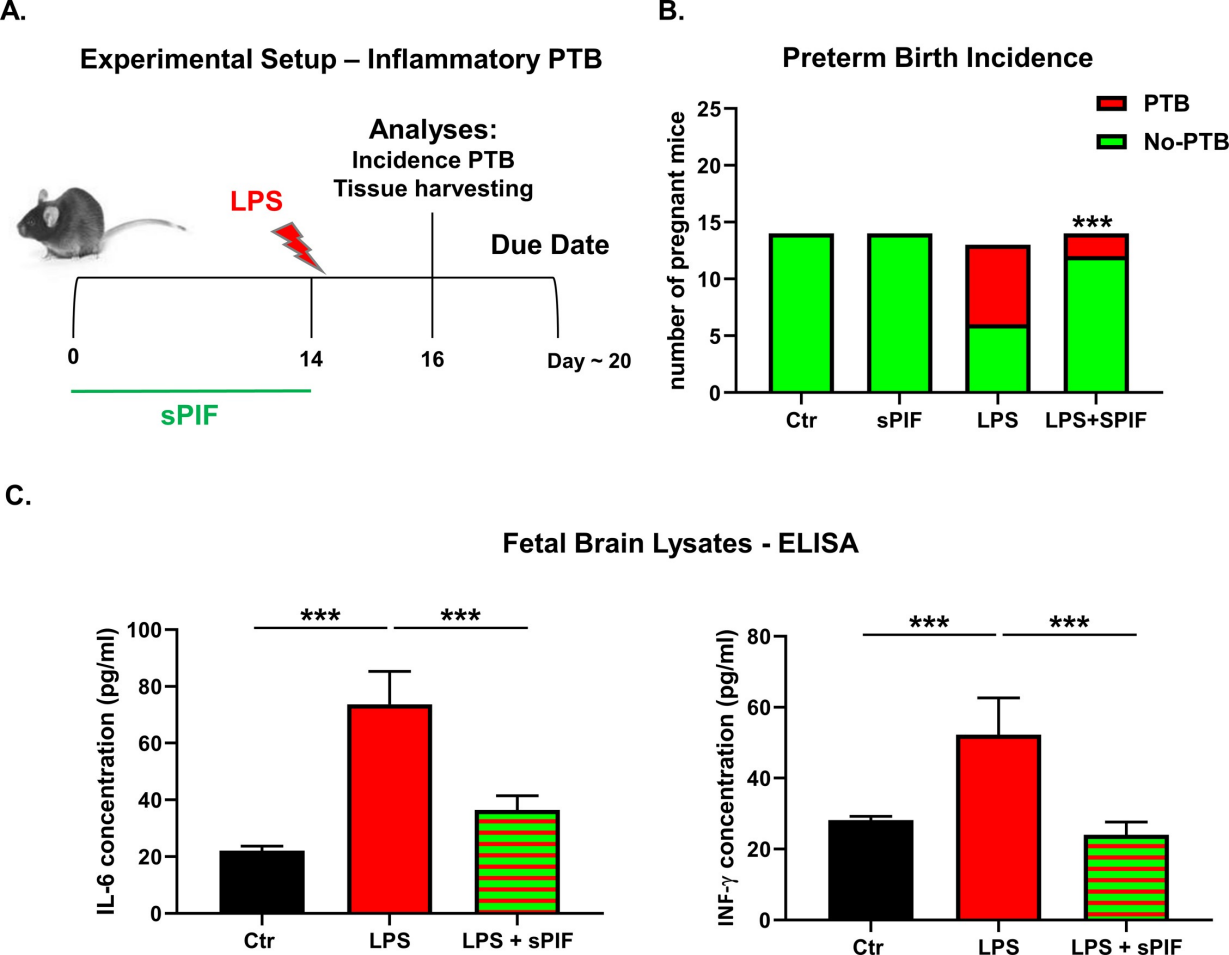
Experimental setup and fetal outcomes after LPS induced insult and synthetic PIF pre-treatment. **A:** Experimental setup: We used 4 experimental groups. Control group received PBS (200 μl/day) on postnatal day 14 (P14) instead of LPS. Synthetic PIF group received synthetic PIF (1μg/g mouse/day) from P0-14 and PBS on P14 (instead of LPS). LPS group received LPS (0.1μg/g mouse) on P14 and LPS/sPIF group received both LPS and synthetic PIF (same as above). **B:** The incidence of PTB. **C:** In fetal brain lysates maternal sPIF pre-treatment reduces pro-inflammatory cytokines IL-6 and INFγ. ***p<0.001. sPIF: synthetic PreImplantation Factor; LPS: Lipopolysaccharides. PTB: Preterm Birth. Data are mean ± SD.

Given PIF`s pro-pregnancy properties in vitro [23] and in vivo [20], we examined synthetic PIF`s properties in a well-established murine model of LPS induced PTB (Fig 1A) [45,46]. This model provides optimal reproductive outcome and it reflects the inflammatory response after a microbial challenge(s) during pregnancy. We administered LPS on day 14 of gestation and analyzed pregnancy outcomes 2 days later, which mimic early inflammatory insult during pregnancy (expected time of delivery day 18–22). Notably, we used sPIF in a maternal prophylactic regime as it reflects potential clinical application (Fig 1A). We detected an increase in prematurity rate after LPS application and sPIF pretreatment reduced this PTB rate significantly (Fig 1B; compare LPS to LPS+sPIF groups). This observation is in line with previous reports of sPIF preventing LPS induced fetal loss [20]. We decided to focus on fetal brains next as an inflammatory insult during pregnancy may result not only in PTB but also in an increased risk of impaired fetal brain development [2,8,9]. We measured IL-6 and INF-γ levels in fetal brain lysates as they impact brain development [5]. For example, IL-6 as a pleiotropic and variably glycosylated cytokine and INF-γ as an immune-regulatory cytokine were reported to impact both PTB and neonatal brain injury [47,48]. Expectantly, we detected increased expressions of IL-6 and INF-γ in fetal brains after maternal LPS application (Fig 1C: compare red to black bars). Maternal sPIF pre-treatment resulted in prevention of pro-inflammatory cytokine (IL-6 and INF-γ) production (Fig 1C: compare green-red striped to red bars). This observation is in line with previous reports of sPIF reducing inflammatory responses in fetal [35,36] and adult brains [33,49,50].

### Synthetic PIF reduces the inflammatory response (in-vivo)

**Fig 2:**
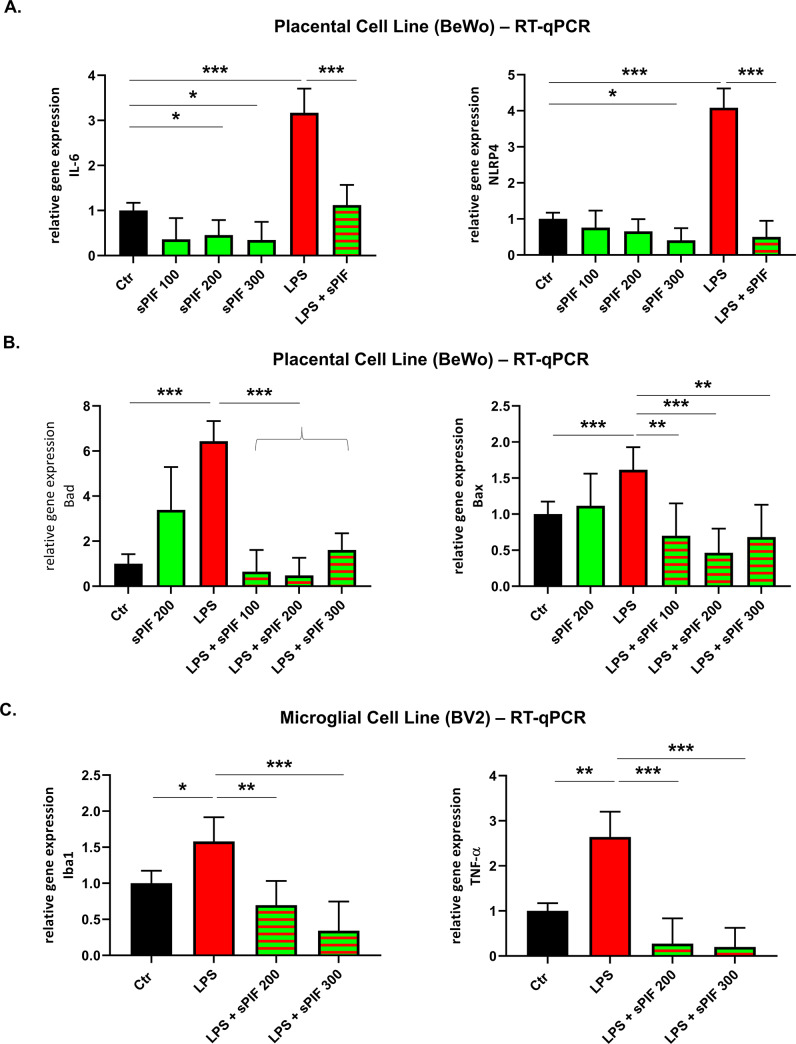
Inflammatory responses. **A** and **B**: Placental cell lines were treated sPIF (200nM), LPS, or LPS + increasing sPIF dose (100–300 nM). We analysed pro-inflammatory **(A)** pro- apoptotic **(B)** genes using RT-qPCR. **C:** Microglial cell lines (BV2) were treated with sPIF (200–300 nM) in the presence of LPS. We analysed pro-inflammatory genes Iba1 and TNF-α. *p<0.05, **p<0.01 and ***p<0.001. sPIF: synthetic PreImplantation Factor; LPS: Lipopolysaccharides. Data are mean ± SD.

Since sPIF modulated inflammation during pregnancy (Fig 1) [20], we decided to test sPIF in challenged placental cells next. We chose to test pro-inflammatory (*IL-6* and *NLRP4*) and pro-apoptotic (*Bad* and *Bax*) genes as they contribute to pregnancy complications such as PTB [5,20,51,52]. We detected moderate changes after sPIF-only treatment (Fig 2A and 2B; compare green to black bars). Therefore, we pretreated cells with LPS (to mimic inflammatory insult) and used sPIF again. LPS application resulted in increased expression of pro-apoptotic (*Bad* and *Bax*) and pro-inflammatory (*IL-6* and *NLRP4*) genes (Fig 2A and 2B; compare red to black bars). Importantly, sPIF reduced these responses significantly (Fig 2A and 2B; compare green-red striped to red bars). Notably, sPIF`s anti-inflammatory and anti-apoptotic effects (Fig 2A and 2B) are in line with previous reports [20,35,36]. Since sPIF modulated the inflammatory responses in the placenta (Fig 2A and 2B) and in fetal brain lysates (Fig 1C), we aimed to test the effect on microglial cells next [35,36]. As seen in Fig 2C, LPS challenge (mimics inflammatory insult) induced the gene expression of ionized calcium binding adaptor molecule 1 (Iba1) and tumor necrosis Factor α (TNF-α). Notably, Iba1 and TNF-α are well-defined markers of inflammation in the brain and sPIF modulated Iba1 previously [33,35,53]. sPIF treatment prevented the LPS induced *Iba1* and *TNF-α* expression (Fig 2C: compare green-red striped to red bars). Together, maternal sPIF pre-treatment reduces the incidence of inflammatory PTB in pregnant animals (Fig 1), which in part is due to reduced inflammatory responses (Fig 2). We aimed to investigate the specific effects in fetal brains next.

### Synthetic PIF prevents inflammatory responses in fetal brain

**Fig 3:**
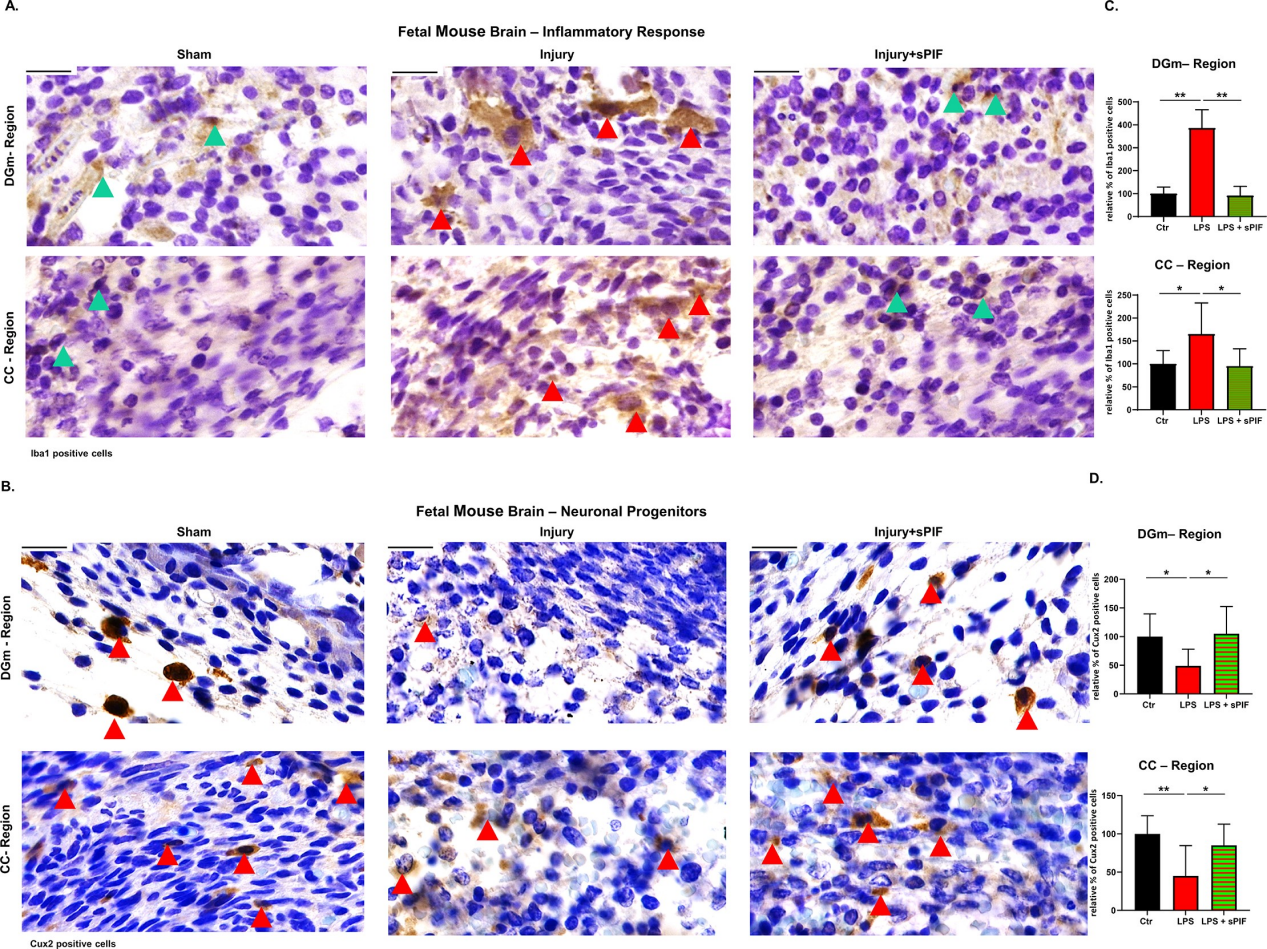
Inflammation and neuronal migration in fetal brains. Representative images of inflammatory markers (A: microglia: Iba1) and neuronal progenitors (B: migrating neurons: Cux2) after LPS-induced insult and maternal sPIF pre-treatment. **A:** We detected increased number of Iba1 positive cells in fetal DGm and CC regions of LPS challenged animals. Maternal sPIF pre-treatment reduced the number of Iba1 positive cells. Green arrowheads indicate examples of amoeboid and red arrows of ramified microglial cells. **B:** We detected reduced number of Cux2 positive cells in fetal DGm and CC regions of LPS challenged animals. Maternal sPIF pre-treatment reduced the loss of Cux2 neurons. Red arrowheads indicate examples of Cux2 positive neurons. **C and D**: Analyses of inflammatory response (Iba1 positive cells) and neuronal migration (Cux2 positive cells) in fetal brains after maternal inflammatory challenge. sPIF: synthetic PreImplantation Factor; LPS: Lipopolysaccharides. DGm: dentate gyrus germinal matrix. CC: cerebral cortex. Data are mean ± SD. Scale bar: 20 μm.

In the central nervous system, microglia (macrophage lineage) represent both the target and source of injury [35,54,55]. Not surprisingly, decreased microglial activation has been associated with reduced cerebral response to injury and restored number of neurons [35,44,56]. The pyramidal neurons are a central part of the mammalian cerebral cortex, which is a six-layered structure [57]. Neurons migrate in a well-defined inside-out fashion. Deep-layers neurons arise and migrate first followed by upper-layers neurons, which are born and migrate later [58]. Notably, in immature brains cortical neurons are especially susceptible to inflammation, injury results in altered cortical development, and Cux2 represents a valid marker of migrating superficial layer neuros [36,59,60]. We evaluated fetal microglial (Iba1 positive cells) and neuronal (Cux2 positive cells) cells after LPS-induced PTB (experimental setup: Fig 1A). We focused on evaluating cortical regions between the rhinal sulcus and the cingulum (CC) and developing dentate gyrus germinal matrix (DGm) as injury in these regions cause distinctive neuropathological alterations [35,39–42]. We detected increased activation of fetal microglia after the inflammatory insult (Fig 3A and 3C; compare Injury to Sham panels and red to black bars), which were abrogated by maternal sPIF pre-treatment (Fig 3A and 3C, compare Injury+sPIF to Injury panels and green-red striped to red bars). Further, in sPIF-treated animals we detected morphological changes in Iba-1 positive microglia. Iba1 positive cells shifted from predominantly amoeboid to ramified state (Fig 3A, compare red to green arrowhead indicated cells). These results are consistent with a view that sPIF reduces cerebral inflammation [35,49]. To evaluate sPIF`s impact on neuronal cells we chose Cux2. Cux2 is a marker of migrating superficial layer neurogenic progenitors [35,36,41,59,60]. We detected decreased number of Cux2 neurons in both cortex and germinal matrix (Fig 3B and 3D; compare Injury to Sham panels and red to black bars). Importantly, sPIF pre-treatment prevented Cux2 neuronal loss (Fig 3B and 3D; compare Injury+sPIF to Injury panels and green-red striped to red bars), which is in line with the reduced inflammatory response (Fig 3A and 3C). These results extend previous reports of PIF`s neuroprotective properties [33,35,36,49,50]. Together, our results provide evidence that maternal sPIF pre-treatment reduces PTB incidence and reduces the inflammatory insult both in the placenta and fetal brain. Given sPIF FAST-Track FDA approval for clinical trial in autoimmune diseases of non-pregnant subjects (clinicaltrials.gov, NCT02239562), prophylactic sPIF treatment in pregnancy can be envisioned.

## Discussion

Our current findings are in line with the notion of PIF`s trophic and protective effects during pregnancy [20,21]. Maternal sPIF pre-treatment provided reduction of inflammatory response to a microbial-like challenge (Figs 1 and 2) to both mother and the fetus. This is important as during PTB the inflammatory insult may effect both the maternal and fetal compartment. Indeed, vaginal dysbiosis may increase susceptibility to pregnancy complications in part by triggering or propagating host inflammatory responses [4,12]. Therefore, the predisposition to PTB may be the result of an overall shift towards an inflammatory environment rather than the presence of individual microbial community [61]. In this case, the modulation of maternal immune-responses, such as sPIF provides, would be an attractive strategy not only in PTB but in other diseases such intrauterine growth restriction or early recurrent pregnancy loss [28,31,62]. Studies addressing fetal growth are being initiated but beyond the scope of this manuscript.

In the fetal brain, sPIF modulated not only the inflammatory response (Figs 1C and 3A) but also neuronal precursors (Fig 3B and 3D). This observation is in line with previous reports of sPIF protecting newborn and adult brains [33,35,36,49,50]. Here, we provide evidence that this effect expands to the fetal period, suggesting a potential effect on neuronal migration/development. Currently, the only interventions known to reduce the burden of brain injury in the term born infants is hypothermia. Hypothermia is associated reduces death and disability in children subjected to perinatal asphyxia significantly [63]. However, approximately half of infants die or develop significant neurological disability despite hypothermia treatment. In the preterm-born population, the antenatal magnesium sulfate prophylaxis (< 30 weeks of gestation) reduces morbidities and mortality rate at 2-years of age. However, magnesium sulfate prophylaxis may result in maternal intoxication and long-term neurological benefits in survivors are lacking [64]. Therefore, sPIF may provide a safe and effective therapy to not only to reduce PTB or fetal loss [20] but may modulate neuronal migration/development in these infants at high-risk [35,36].

Finally, we acknowledge that the current study has some minor limitation. The primary limitation of the study is the lack of functional outcomes in the offspring. Although we did not provide functional testing, we show modulation of neuronal progenitors and microglial cells of the fetuses and our results are in line with previous evidence of sPIF neuroprotective properties [33,35,36,49,50]. Further studies will address sPIF`s impact during fetal brain development. We further acknowledge that we didn`t provide detailed insights to potential pathways involved in immune modulation or neuronal regulation. We provide evidence that sPIF prevents PTB efficiently and additional analyses including response not only bacterial but also viral challenges are ongoing but beyond the scope of this manuscript.

In conclusion, we provide evidence that sPIF is a potential therapeutic (targeted prophylaxis) in inflammatory induced PTB. These results are in line with sPIF`s efficacy previously observed in several preclinical models of immune disorders [20,32–38]. Importantly, this is the first description of sPIF restoring PTB related morbidities such as fetal brain development. Given that sPIF finished first-in-human Phase Ib clinical trial (NCT02239562), sPIF therapy in pregnancy disorders such as preeclampsia, early pregnancy loss, and fetal loss can be envisioned [20,21,27].
